# The Role of Hydrogen for *Sulfurimonas denitrificans’* Metabolism

**DOI:** 10.1371/journal.pone.0106218

**Published:** 2014-08-29

**Authors:** Yuchen Han, Mirjam Perner

**Affiliations:** Molecular Biology of Microbial Consortia, University of Hamburg, Biocenter Klein Flottbek, Hamburg, Germany; Instituto de Tecnologica Química e Biológica, UNL, Portugal

## Abstract

*Sulfurimonas denitrificans* was originally isolated from coastal marine sediments. It can grow with thiosulfate and nitrate or sulfide and oxygen. Recently sequencing of its genome revealed that it encodes periplasmic and cytoplasmic [NiFe]-hydrogenases but the role of hydrogen for its metabolism has remained unknown. We show the first experimental evidence that *S. denitrificans* can indeed express a functional hydrogen uptake active hydrogenase and can grow on hydrogen. In fact, under the provided conditions it grew faster and denser on hydrogen than on thiosulfate alone and even grew with hydrogen in the absence of reduced sulfur compounds. In our experiments, at the time points tested, the hydrogen uptake activity appeared to be related to the periplasmic hydrogenase and not to the cytoplasmic hydrogenase. Our data suggest that under the provided conditions *S. denitrificans* can grow more efficiently with hydrogen than with thiosulfate.

## Introduction

*Epsilonproteobacteria* have been recognized as a physiologically versatile microbial lineage which appears to be relevant for element cycling in ubiquitous environments (cf. [1]). Many *Epsilonproteobacteria* have a chemolithoautotrophic lifestyle, where the oxidation of reduced sulfur compounds or hydrogen can be coupled to oxygen or nitrate reduction or where hydrogen can be oxidized with elemental sulfur as electron acceptor (cf. [1]). Among the *Epsilonproteobacteria, Sulfurimonas* is a genus of which currently only a few cultured representatives are known including *S. autotrophica*, *S.* sp. CVO, *S.* sp. YK-1, *S. paralvinella*, *S. gotlandica,* and *S. denitrificans* [2]–[7]. They have been isolated from a vast array of (sulfidogenic) habitats including marine sediments [5], deep-sea hydrothermal vent habitats [2], [3], pelagic redoxcline [4] and oil fields [6], [7].

*S. autotrophica* was described as mesophilic, sulfur-, sulfide- and thiosulfate-oxidizing chemolithoautotroph [2]. It does not grow on hydrogen [2] and lacks hydrogen uptake activity [8]. *S.* sp. CVO grows only on sulfide and elemental sulfur with acetate as well as CO_2_ as sole carbon source [7]. *S.* sp. YK-1 utilizes sulfide, elemental sulfur, thiosulfate as well as hydrogen [6]. *S. paralvinella* is a mesophilic sulfur- and hydrogen-oxidizing chemolithoautotroph [3] and its crude extracts exhibit hydrogen uptake activity [8]. Only recently the chemolithoautotrophic *S. gotlandica* was isolated, which can utilize reduced sulfur species (elemental sulfur, sulfide and thiosulfate) as well as hydrogen [4]. Although *S. denitrificans* was initially grown on reduced sulfur compounds and characterized as a thiosulfate oxidizer [5], recent sequencing of its genome revealed periplasmic and cytoplasmic [NiFe]-hydrogenases [9]. Based on the information gained through genome sequencing, *S. denitrificans* could be successfully cultivated with hydrogen as the electron donor (cf. [9]), but hydrogen uptake activities are still not available and the role that hydrogen plays for its growth remains unknown.

Although we know that certain *Sulfurimonas* species can utilize hydrogen [4], [6], [8], in the past this genus has been referred to as being a sulfur-oxidizing lineage [1]. In some hydrogen-rich hydrothermal environments 16S rRNA genes of *Sulfurimonas* species have been reported to be particularly abundant [10] and environmental hydrogenase gene sequences resembling those of *S. denitrificans* have been detected [11]. In summary, this data suggests that hydrogen may be more important for the energy metabolism of *S. denitrificans* and possibly also for other *Sulfurimonas* species than currently thought. Since several *Sulfurimonas* strains can utilize sulfur compounds as well as hydrogen, we here investigate the role that hydrogen plays for growth and metabolism in *Sulfurimonas* and use *S. denitrificans* as a model organism.

## Materials and Methods

### Bacterial strains and growth conditions

Bacterial strains and plasmids are listed in Table 1. *E. coli* strains were cultured in Luria–Bertani (LB) broth at 37°C. *Shewanella oneidensis ΔhyaB* derived stains were grown at 28°C, either aerobically in LB broth or anaerobically in fresh water medium (modified according to Lovley, supplemented with 15 mM pyruvate and 15 mM fumarate) [12] with H_2_/CO_2_ (80%/20%, 1 atm) (Westfalen AG, Münster, Germany) in the headspace. When required, antibiotics were used at the following concentrations: kanamycin 20 µg ml^−1^; gentamycin 10 µg ml^−1^.

**Table 1:** Strains and plasmids used in this study.

Strain or plasmid	Relevant features	References/source
***E. coli***		
DH5α	Host strain for plasmid constructions, *fhuA2 Δ(argF-lacZ) U169 phoA glnV44 Φ80 Δ(lacZ) M15 gyrA96 recA1 relA1 endA1 thi-1 hsdR17*	New England Biolabs
***S. oneidensis***		
*ΔhyaB*	MR-1, *hyaB*::Gm^r^	Provided by Nicolas Rychlik
***S. denitrificans***		
DSM-1251	Wild type	[5]
**Plasmids**		
pBBR1MCS-2	Broad-host-range cloning vector, Km^r^	[20]
pBBR1MCS2_Suden*hyd*B	pBBR1MCS-2::*hyd*B (Suden_1435), Km^r^	This study
pRK2013	Helper plasmid for the triparental conjugation, Km^r^	[21]
pGEM-T	Cloning vector with T overhangs, *lacZ*, Ap^r^	Promega

*S. denitrificans* was routinely grown in serum bottles in DSMZ medium 113 with 18.8 mM thiosulfate at 25°C under anaerobic conditions with H_2_/CO_2_ (80%/20%, 1 atm) in the headspace. These cultures served as pre-cultures (1 week growth) for further experiments with different medium. To test cell growth of *S. denitrificans* under anaerobic conditions, 1 mL of the pre-culture was transferred to fresh medium (125 mL). Four different incubation conditions were tested: (i) H_2_/CO_2_ (80/20%) with thiosulfate, (ii) H_2_/CO_2_ (80/20%) without thiosulfate, (iii) N_2_/CO_2_ (80%/20%, 1 atm) (Westfalen AG, Münster, Germany) with thiosulfate and (iv) N_2_/CO_2_ (80%/20%, 1 atm) without thiosulfate. To monitor the growth of *S. denitrificans*, the amounts of total protein in 1 mL cell culture at each indicated time point were measured (three times) according to Bradford as previously described [13], [14]. The experiments were performed three times and exhibited the same general trend, i.e. all H_2_/CO_2_ experiments with thiosulfate exhibited faster growth initially, but a lower growth yield overall relative to the H_2_/CO_2_ experiments without thiosulfate. For selected time points p-values were calculated using the student’s t-test.

### Determination of nitrate and thiosulfate

*S. denitrificans* was grown under the four different conditions as described above. At each indicated time point 1 mL culture was harvested by centrifugation. Supernatants were kept for nitrate and thiosulfate measurements, and cell pellets for total protein measurements (see above). For the measurement of nitrate in the medium the supernatant was diluted 1∶20 with Milli-Q water and from this dilution 25 µL was taken for the measurement. Nitrate concentration was determined by high-performance liquid chromatography (HPLC) via ion-pair chromatography with a LiChrospher RP-18 column (5 µm; 125 by 4 mm; Merck KGaA, Darmstadt, Germany) [15] and UV detection in an automated system (HPLC-System LaChrom Elite, VWR International GmbH, Darmstadt, Germany). For the measurement of thiosulfate in the medium the supernatant was diluted 1∶250 with Milli-Q water and from this dilution 250 µL was taken for the measurement. The amount of thiosulfate was determined by measuring the discoloration of methylene blue spectrophotometrically at 670 nm [16]. The measurements at the different time points were performed with three independent cultures.

### *In vivo* hydrogen measurements

For *in* *vivo* hydrogen measurements *S. denitrificans* was grown anaerobically with H_2_/CO_2_ (80%/20%, 1 atm) as described above with thiosulfate (18.8 mM) or without thiosulfate. Controls were set up which consisted of all medium ingredients required for *S. denitrificans* cultivation but without inoculated bacteria. For every hydrogen measurement 0.25 mL of the headspace was extracted, diluted 1∶2,300 with nitrogen (5.0; Westfalen AG, Münster, Germany) and from this dilution 2 mL of gas mixture was injected into the gas chromatograph (GC; Thermo Fischer Scientific Inc, Waltham, MA, USA). The amount of hydrogen in the headspace was measured by pulsed discharge ionization detector in the GC. ShinCarbon ST 100/120 (Restek, Bellefonte, PA, USA) was used as a column and helium as a carrier gas. These experiments were performed in triplicate. Statistics were performed using the student’s t-test.

### Reverse transcription quantitative PCR (RT-qPCR)

*S. denitrificans* was grown anaerobically with H_2_/CO_2_ (80%/20%, 1 atm) and thiosulfate as described above. Subsamples (10 mL) of the cultures were taken after three, six and twelve days of incubation. The total RNA was isolated with Presto Mini RNA Bacteria Kit (Geneaid Biotech, Taiwan). DNA was removed by using RTS DNase Kit (MO BIO Laboratories, Carlsbad, CA, USA). cDNAs were synthesized from 700 ng RNA with random primers by using SuperScript VILO MasterMix (Life Technologies, Carlsbad, CA, USA) in a final volume of 20 µL. A control reaction lacking reverse transcriptase was performed. PCR amplifications were carried out with cDNA product as template and specific primers (5′-ATATCGTAATGGCGGCAGAG-3′ and 5′-CATCAGGTCCAACAGTATCG-3′ for *hydB*, Suden_1435, the large subunit of the periplasmic hydrogenase; 5′-TGCGGAATATGTGGACATGC-3′ and 5′-ACTATCGCGTAAGAGGTGTG-3′ for Suden_1437, the large subunit of cytoplasmic hydrogenase; 5′-TGGATTCGCCAAGCAATCTC-3′ and 5′-GCGCCCATCATCTTCACTTC-3′ for *rpoD*, Suden_1105, RNA polymerase sigma factor) [9] by using SYBR Select Master Mix for CFX (Life Technologies, Carlsbad, CA, USA). The relative gene expression was calculated with Bio-Rad CFX Manager (Bio-Rad Laboratories, Hercules, CA, USA) and related to the gene expression of *rpoD* (housekeeping sigma factor). The experiments were conducted three times and the student’s t-test was performed for statistics.

### Hydrogenase activity assay

*S. oneidensis* strains and *S. denitrificans* were grown anaerobically with H_2_/CO_2_ (80%/20%, 1 atm) as described above. The hydrogenase activity assays were performed in three parallels. Subcellular fractionations were performed in an anaerobic chamber (Coy Laboratory Products, Grass Lake, MI, USA) as previously described [17]. The assays were performed as described before [17]–[19] with oxidized methyl viologen (MV, Sigma-Aldrich). In the presence of an active hydrogenase the MV is reduced to MV^+^ (blue form), which was quantified spectrophotometrically at 602 nm. All measurements were performed at 25°C and the hydrogen uptake activity was calculated by using an extinction coefficient of 5,401 M^−1^ cm^−1^ (personal communication, Nicolas Rychlik). The student’s t-test was performed for assessing the statistical relevance of the findings.

### Construction of expression vectors

The 1.7-kb Suden_1435 (*hydB*, the large subunit of the periplasmic hydrogenase) [9] was amplified from the genomic DNA of *S. denitrificans* by using the primers Suden1435F (5′-ATGTCAAAAAGAGTAATAGTAGA-3′) and Suden1435R (5′-TTAAATAGTGCATCCGCCATA-3′). The fragment was ligated with pGEM-T (Promega, Mannheim, Germany), restricted with SacI and SacII, and religated to SacI/SacII restricted pBBR1MCS-2 [20]. The sequence and the direction of gene Suden_1435 were confirmed by sequencing (Eurofins MWG operon, Hamburg, Germany). The plasmids were transferred into *S. oneidensis ΔhyaB* by tri-parental conjugation with the helper plasmid pRK2013 [21].

## Results and Discussion

### Growth of *S. denitrificans* on hydrogen and *in* *vivo* hydrogen consumption

**Figure 1:**
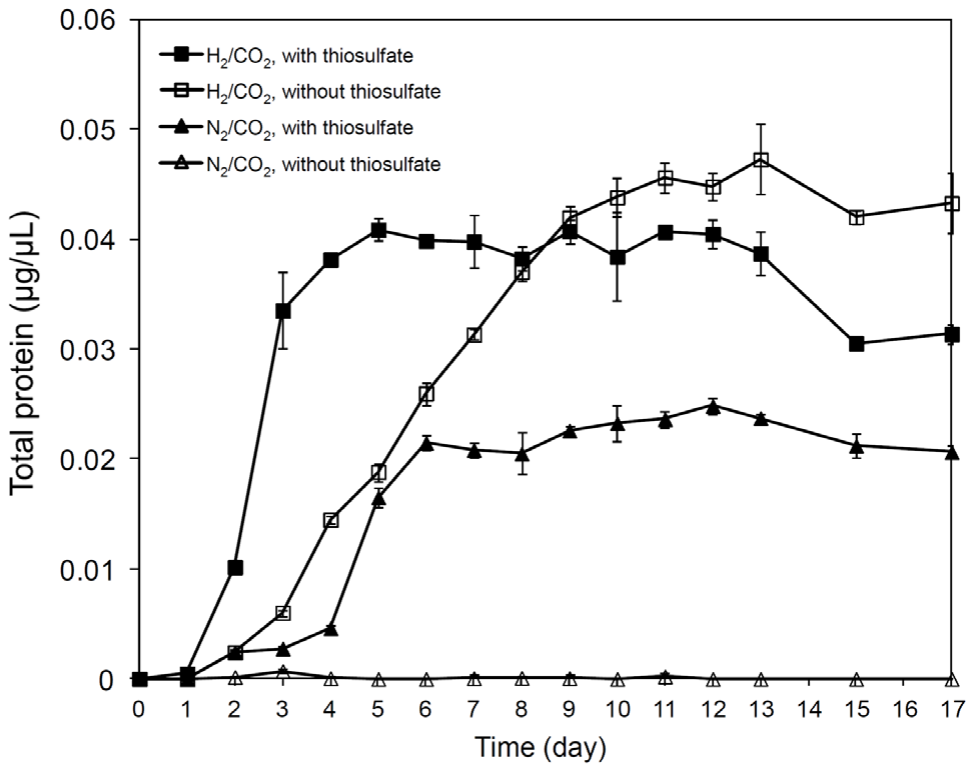
Growth of *S. denitrificans* DSM-1251 under different cultivation conditions. *S. denitrificans* DSM-1251 cells were grown in DSMZ medium 113 containing thiosulfate (18.8 mM, filled marker) or without thiosulfate (open marker). The headspace was completely exchanged with H_2_/CO_2_ (80%/20%) (square) or with N_2_/CO_2_ (80%/20%) (triangle). Error bars indicate the standard deviations from measurements of three cultures.

**Figure 2:**
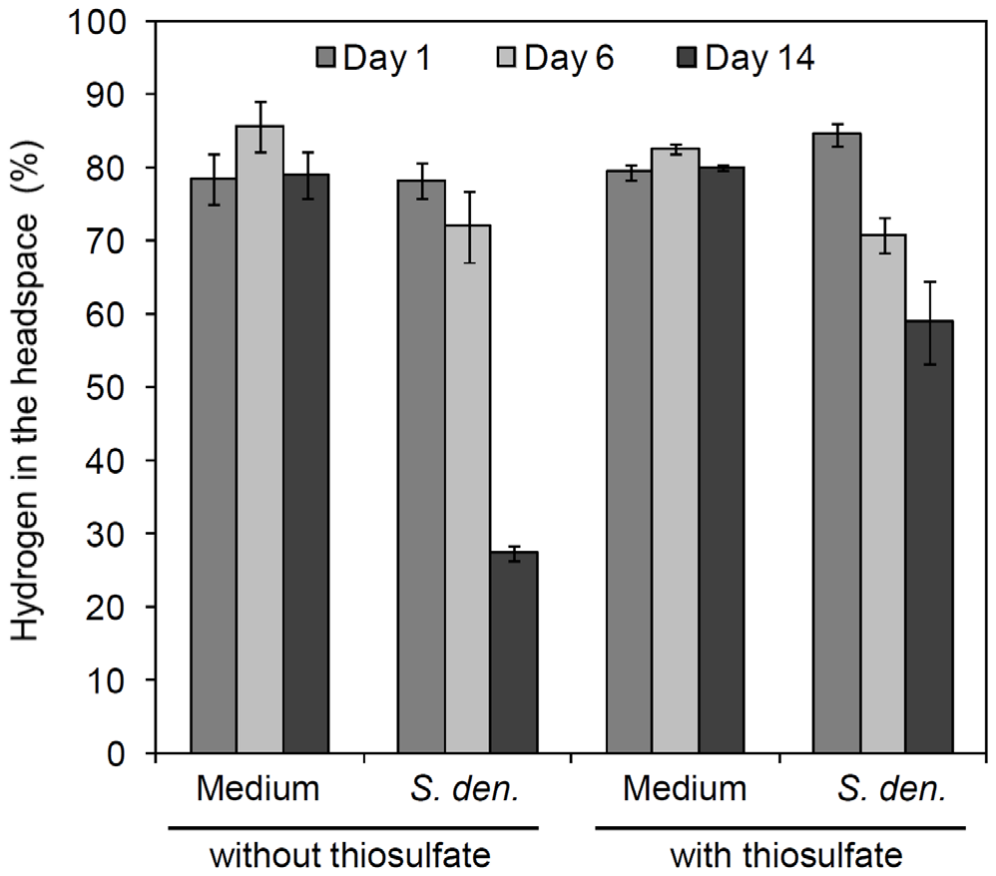
*In vivo* hydrogen consumption in *S. denitrificans* DSM-1251. *S. denitrificans* DSM-1251 cells were grown anaerobically in DSMZ medium 113 either with thiosulfate (18.8 mM) or without thiosulfate. On the first day of inoculation, the headspace of each sample was exchanged with H_2_/CO_2_ (80%/20%). The H_2_ concentration in the headspace was measured with gas chromatography. A control experiment was set up to exclude hydrogen leakage from the vessels. Medium = control, medium without inoculated *S. denitrificans* DSM-1251; *S. den.* = inoculation with 0.5 mL of *S. denitrificans* DSM-1251 pre-culture at day 1. Error bars of “Medium” and “*S. den.*” indicate the standard deviations from three independent experiments.

To monitor the growth of *S. denitrificans* under different conditions, namely (i) H_2_/CO_2_ (80%/20%) with thiosulfate (18.8 mM), (ii) H_2_/CO_2_ (80%/20%) without thiosulfate, and as controls (iii) N_2_/CO_2_ (80%/20%) with thiosulfate (18.8 mM) and (iv) N_2_/CO_2_ (80%/20%) without thiosulfate, the amount of total protein was recorded at different growth phases. *S. denitrificans* cells grown anaerobically in DSMZ medium 113 grew significantly denser (p-value<0.001) when only hydrogen was present relative to when only thiosulfate was offered (Figure 1, Table S1) clearly indicating that besides thiosulfate oxidation, hydrogen oxidation is also used as an energy source in these cultures (Figure 1). *In vivo* hydrogen consumption measurements with *S. denitrificans* confirmed that hydrogen was being actively consumed when the headspace of the culture was filled with hydrogen, regardless whether thiosulfate was present or not (Figure 2).

**Figure 3:**
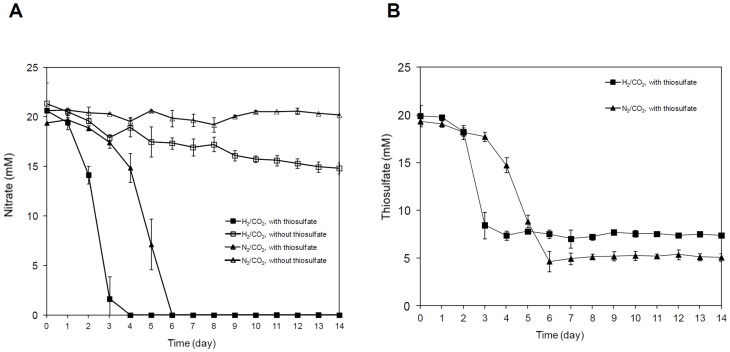
Nitrate (A) and thiosulfate (B) consumption of *S. denitrificans* DSM-1251 under different cultivation conditions. *S. denitrificans* DSM-1251 cells were grown in DSMZ medium 113 containing thiosulfate (18.8 mM, filled marker) or without thiosulfate (open marker). The headspace was completely exchanged with H_2_/CO_2_ (80%/20%) (square) or with N_2_/CO_2_ (80%/20%) (triangle). Error bars indicate the standard deviations from measurements of three cultures.

Based on protein contents, cells which were fed with hydrogen and thiosulfate grew significantly faster within the first three days (0.011 µg protein/µl culture per day) (p-value 0.003) than those fed with hydrogen and lacked thiosulfate (0.002 µg protein/µl culture per day) and reached maximum total protein concentrations of 0.041±0.001 and 0.047±0.003 µg/µl, respectively (Figure 1, Table S1). However, cells in medium with hydrogen in the headspace but without thiosulfate grew significantly denser overall (p-value 0.002) (Table S1). The initial rapid growth of cells grown on hydrogen and thiosulfate during the first three days correlated with a steep decrease in nitrate (19 mM was consumed) (Figure 3A) and thiosulfate (13 mM was consumed) (Figure 3B). By the fourth day all the nitrate was used up, thiosulfate oxidation ceased and cells reached the stationary phase by the fifth day. In contrast, cells grown on hydrogen but without thiosulfate exhibited less rapid growth and only consumed ∼6.5 mM nitrate (over 14 days) and cells entered the stationary phase late, namely after 11 days. Also, significantly less hydrogen was consumed in cultures where thiosulfate was supplemented (30% of hydrogen consumed after 14 days of cultivation, i.e. ∼11 mM) than in those lacking thiosulfate (65% of hydrogen consumed after 14 days of cultivation, i.e. 23 mM) (p-value 0.001) (Figure 2, Table S2).

Taken together, these data demonstrate that hydrogen can indeed serve as an electron donor for the growth of *S. denitrificans* and that under the conditions provided it can grow faster and denser with hydrogen than with thiosulfate. In contrast to experiments with *S. paralvinella*, where hydrogen is only consumed when reduced sulfur compounds are available [3], *S. denitrificans* can grow with hydrogen without reduced sulfur sources being present.

### Hydrogenase transcript in *S. denitrificans*

**Figure 4:**
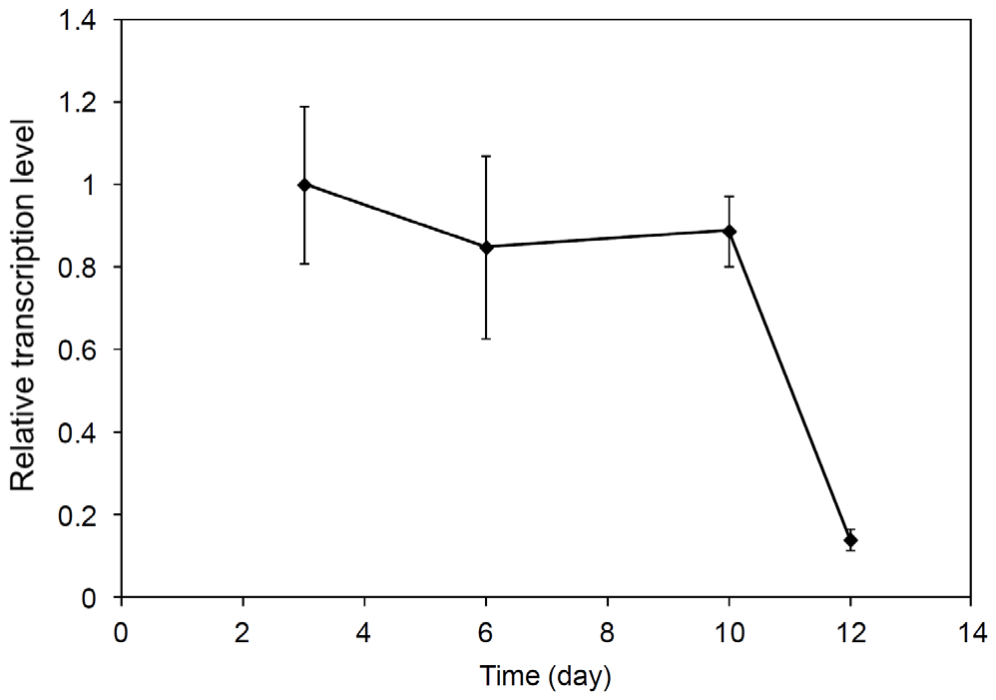
Transcript of *hydB* (Suden_1435) in *S. denitrificans* DSM-1251. *S. denitrificans* DSM-1251 cells were grown in DSMZ medium 113 with thiosulfate (18.8 mM) and H_2_/CO_2_ (80%/20%) in the headspace. The relative transcription levels of *hydB* were quantified by RT-qPCR and normalized to the reference gene *rpoD* (housekeeping sigma factor). The transcription level of *hydB* after 3 days was set as 1. Error bars denote standard deviations from three independent experiments.

Genome sequencing of *S. denitrificans* revealed hydrogenases on its genome [9]: it encodes the *hyd* operon with one cytoplasmic hydrogenase, one membrane-bound hydrogenase, and accessory proteins for the hydrogenase assembly. The cytoplasmic hydrogenase has been posited to reduce electron acceptors with very negative redox midpoint potentials and may provide low-potential electrons to the rTCA [9]. If this proves true the need for a reverse electron transport is circumvented because reducing power can be generated directly and as a consequence the efficiency of growth is increased [9]. When cells were grown in the presence of hydrogen and thiosulfate, transcripts of the cytoplasmic hydrogenase were recovered (demonstrated by RT-PCR, data not shown). In many proteobacteria such as *Wolinella succinogenes* and *Shewanella oneidensis*, the periplasmic [NiFe]-hydrogenase serves as the major hydrogen uptake hydrogenase (Group I hydrogenase) [22]. This group of hydrogenases is capable of supporting growth with hydrogen as an energy source. They catalyze the oxidation of hydrogen to protons and electrons. The oxidation of hydrogen is coupled to the reduction of electron acceptors with the recovery of energy in the form of a proton motive force [23]. In *S. denitrificans* Suden_1435 encodes for the catalytic large subunit (HydB) and Suden_1436 for the small subunit (HydA) of the group I periplasmic [NiFe]-hydrogenase [9]. Our RT-qPCR illustrated that Suden_1435 (*hydB*) is indeed transcribed in *S. denitrificans* (Figure 4). RT-qPCR performed on the total RNA from *S. denitrificans* grown in the presence of hydrogen and thiosulfate further demonstrated that *hydB* was transcribed during the first 10-days of growth but then its transcription level decreased significantly (p-value<0.005) (Figure 4). The transcription level appeared to follow the general trend of growth of *S. denitrificans* (Figure 1, Figure 4). When *S. denitrificans* was in the exponential growth phase, the transcription level of the hydrogenases was highest and started to decline when the stationary growth phase was reached.

### Hydrogenase activity of *S. denitrificans* HydB

**Figure 5:**
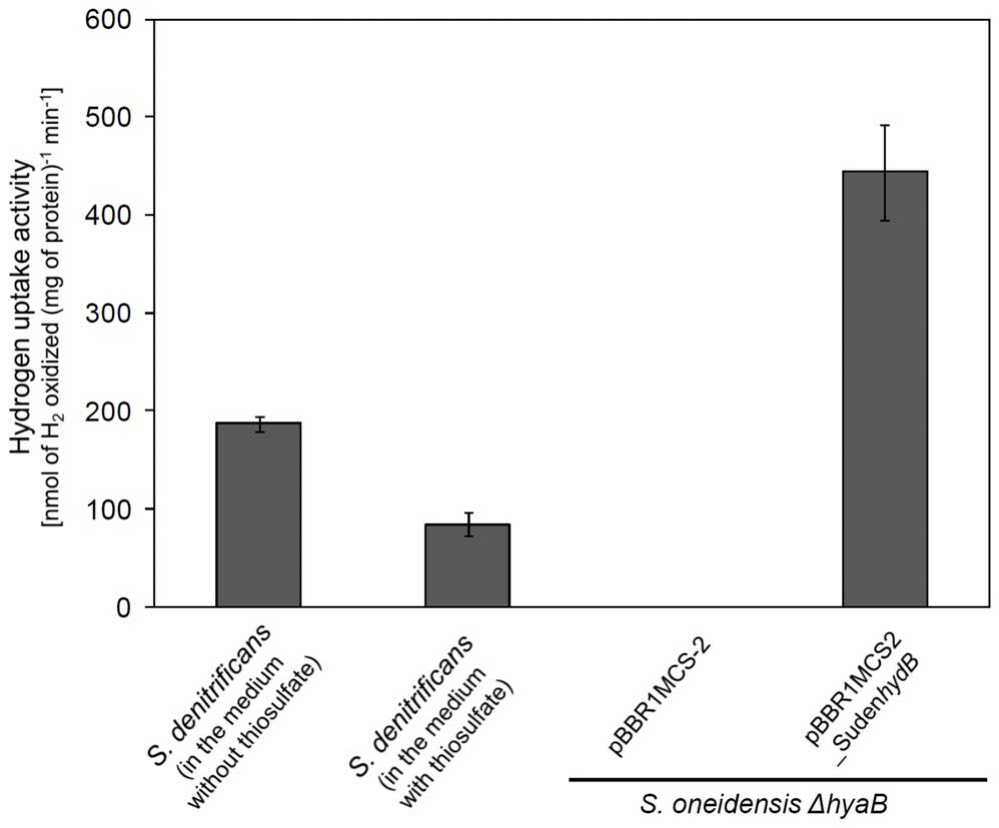
Hydrogen uptake activity of the native and recombinant hydrogenase (HydB) from *S. denitrificans*. Hydrogen uptake activity was measured in the membrane fractions of *S. denitrificans* grown without and with thiosulfate (18.8 mM) and for the recombinant version of the large subunit (HydB). The large subunit of the periplasmic hydrogenase (*hydB*) was cloned into the vector pBBR1MCS-2 and expressed heterologously in *S. oneidensis ΔhyaB*. The empty vector in *S. oneidensis ΔhyaB* was used as negative control. Error bars denote standard deviations from three independent measurements.

To study the hydrogen uptake activity *in* *vitro*, we performed the activity assay with the soluble and membrane fractions of *S. denitrificans*. No hydrogen uptake activity was detected in the soluble fraction at the sampled time point (data not shown), although a cytoplasmic hydrogenase is encoded on the *S. denitrificans* genome [9] and transcripts of the cytoplasmic hydrogenase were recovered. The hydrogen uptake activity in the membrane fraction was 187±7 nmol of H_2_ oxidized (mg of protein)^−1^ min^−1^ (Figure 5), when *S. denitrificans* was grown with hydrogen but without thiosulfate in the medium. This is roughly 10-fold lower than hydrogen uptake activity measured in *S. paralvinella* [8]. However, the hydrogen uptake activity from *S. denitrificans* was higher than the hydrogen uptake activity detected in other proteobacteria, e.g., *Gamaproteobacterium Hydrogenovibrio marinus* [49 nmol of H_2_ oxidized (mg of protein)^−1^ min^−1^] and *Betaproteobacterium Cupriavidus metallidurans* [previously known as *Alcaligenes eutrophus*, 42 nmol of H_2_ oxidized (mg of protein)^−1^ min^−1^] at 37°C (in the same buffer we used) [18]. When *S. denitrificans* was grown in the medium with thiosulfate, an activity of 85±12 nmol of H_2_ oxidized (mg of protein)^−1^ min^−1^ was detected in the membrane fraction (Figure 5). This activity is significantly lower (p-value<0.001, roughly 2-times, Table S2) than the hydrogen uptake activity we measured for cells grown with hydrogen but without thiosulfate. These results are in line with the lower *in* *vivo* hydrogen consumption for *S. denitrificans* grown with thiosulfate (Figure 2) and together with thiosulfate consumption measurements (Figure 3B) suggest that if thiosulfate is present, under the provided conditions, *S. denitrificans* oxidized thiosulfate besides hydrogen which appears to result in lower hydrogen requirement.

To ensure that the periplasmic hydrogenase is responsible for the hydrogen uptake activity we cloned the large subunit of the periplasmic hydrogenase (*hydB*) in the vector pBBR1MCS-2. Recombinant expression of *hydB* demonstrated an activity of 444±49 nmol of H_2_ oxidized (mg of protein)^−1^ min^−1^ in the membrane fraction (Figure 5). This was in fact twice as high as the non-recombinant expression in *S. denitrificans* grown without thiosulfate, but can be explained by the copy number of the plasmid: pBBR1MCS-2 is a medium-copy-number plasmid [24] and thus several copies of the plasmid are present in the cell expressing the hydrogenase. In summary, these results indicate that the periplasmic hydrogenase is most likely if not solely responsible for the measured hydrogen uptake activity in the crude extracts.

### The role of hydrogen for *S. denitrificans* metabolism

*S. denitrificans* clearly exhibits different growth behaviors, hydrogen and nitrate consumption and hydrogen uptake activity depending on whether the cells are grown on hydrogen with or without thiosulfate. This is expressed in rapid growth, initially, but overall lower cell numbers, higher nitrate consumption (to the point of nitrate limitation), but lower hydrogen consumption (two-fold) and lower hydrogen uptake activity (two-fold) when thiosulfate is supplemented to the hydrogen medium. The reaction 5H_2_+2NO_3_^−^+2H^+^>>6H_2_O+N_2_ yields −959 kJ/reaction whereas 5S_2_O_3_^2−^+8NO_3_^−^+H_2_O>>10SO_4_^2−^+2H^+^+4N_2_ yields -3926 kJ/reaction [25]. Hence, roughly four times more energy can be gained if thiosulfate is oxidized, but then also four times more nitrate is consumed relative to the reaction involving hydrogen. Organisms without the need of a reverse electron transport require 1060 kJ catabolic energy to fix one mol of carbon in biomass [26]. Assuming that with and without thiosulfate in the hydrogen medium the cytoplasmic hydrogenase catalyzes the reduction of NAD with hydrogen, thereby creating reducing power directly, theoretically considerably more biomass can be synthesized when thiosulfate is oxidized (3.7 mol carbon per five molecules thiosulfate) than when hydrogen is oxidized (0.9 mol carbon per five molecules hydrogen) with nitrate. This likely explains why the cells grown on thiosulfate grow faster initially than the cells grown on hydrogen without thiosulfate.

In the growth experiments with hydrogen and thiosulfate, according to the stoichiometry, the 13 mM consumed thiosulfate, if coupled to nitrate reduction, would theoretically account for the consumption of 19 mM nitrate, which is the measured amount of nitrate that was utilized in this experiment (Figure 3A). This would suggest that no nitrate reduction coupled hydrogen oxidation occurs, although 11 mM of hydrogen was used (Figure 2). While some of this hydrogen is likely essential to directly reduce NAD to produce reducing power directly, the hydrogen uptake activity of the periplasmic hydrogenase (although two-fold lower than when cells are grown without thiosulfate) (Figure 5) strongly suggests that energy generation in cells under these conditions is likely also linked to the build up of a proton motive force during hydrogen oxidation. Two scenarios could explain this discrepancy: based on *S. denitrificans* available genome information, genes encoding proteins that may be involved in the reduction of sulfur compounds including thiosulfate have been identified [9], which may account for some of the used thiosulfate. Hence, some of the nitrate may indeed be available for hydrogen oxidation coupled nitrate reduction. Hydrogen oxidation may also be coupled to sulfate reduction. *S. denitrificans* has an operon that encodes proteins of sulfate reduction [9]. However, the energy yield of 4H_2_+ SO_4_^2-^ +2H^+^ >> H_2_S+4H_2_O is considerably lower (154 kJ/reaction, [25]) than for nitrate reduction coupled hydrogen oxidation. However, based on the rapid cell growth in cultures with hydrogen and thiosulfate relative to cultures without thiosulfate we believe that thiosulfate oxidation represents the major mode of energy generation in these hydrogen incubations supplemented with thiosulfate and that under these conditions most of the consumed hydrogen is related to creating reducing power directly.

When no thiosulfate is present, the cells revert to generating energy from hydrogen oxidation and twice as much hydrogen is consumed (Figure 2) and hydrogen uptake activity is doubled (Figure 5). In the experiments where cells were grown on hydrogen without thiosulfate, 6.5 mM nitrate was consumed when cells reached the stationary phase, which, according to the stoichiometry, could fuel the oxidation of ∼16 mM hydrogen. In these cultivations around 23 mM hydrogen was used leaving theoretically ∼7 mM hydrogen unaccounted for. As suggested above some of this hydrogen is likely used to circumvent the need for reversed electron transport, but the two-fold higher hydrogen uptake activity in these cultures also hint to the larger requirement of energy generation through hydrogen oxidation. Under these conditions some of the hydrogen oxidation may also be coupled to sulfate reduction - it has been postulated that the operon structure encoding the proteins for sulfate reduction can be turned on or off depending on the concentration of reduced inorganic sulfur species in the environment [9]. The lower energy yield through hydrogen oxidation coupled to nitrate or sulfate reduction relative to thiosulfate oxidation explains the slower growth yields. Since these cells are not limited by nitrate, as those in the hydrogen incubations with thiosulfate, growth can proceed to eventually reaching higher biomass than in the cultures with thiosulfate, which started being limited by nitrate after 3 days.

Finally, based on our experiments we cannot state how much of the consumed nitrate is related to nitrate reduction coupled to thiosulfate oxidation versus hydrogen oxidation or how much hydrogen is used in the experiments for energy generation through nitrate or sulfate reduction or for production of reducing power. Further experiments will be needed to address these questions in detail.

## Conclusion

Our study provides the first experimentally shown evidence that *S. denitrificans* can express a functional hydrogen uptake hydrogenase and use hydrogen as electron donor. In fact, in our experiments *S. denitrificans* grew faster and denser on hydrogen than on thiosulfate alone. The cells could also gain energy through the oxidation of hydrogen in the absence of reduced sulfur compounds. Although thiosulfate oxidation yields more energy per reaction than hydrogen oxidation does, it also uses four times more nitrate than when hydrogen oxidation is coupled to nitrate reduction. Thus, when the cells oxidize thiosulfate they become limited by nitrate quicker than if they oxidize hydrogen. The many environmental sequences available in the databases resembling those of *S. denitrificans* may in fact not be solely associated with sulfur metabolism but may also represent microbes that are involved in hydrogen turnover. In fact, in the environment under nitrate limiting conditions hydrogen oxidation may be a favored metabolism.

## Supporting Information

Table S1 Growth properties and significant differences in distinct culture experiments. *S. denitrificans* were incubated under different conditions as listed in Figure 1. For selected time points p-values were calculated using the student’s t-test.
(PDF)

Table S2 Significant differences of growth, hydrogen consumption and hydrogen uptake activity for cultures grown without thiosulfate (+H_2_−S_2_O_3_^2−^) and cultures grown with thiosulfate (+H_2_+S_2_O_3_^2−^). *S. denitrificans* were grown without thiosulfate (+H_2_−S_2_O_3_^2−^) and with thiosulfate (+H_2_+S_2_O_3_^2−^), the growth, H_2_ consumption and H_2_ uptake activity are shown in Figure 1, Figure 2 and Figure 5, respectively. Statistics were performed using the student’s t-test.
(PDF)
