# Supplementary material for: The Role of Hydrogen for Sulfurimonas denitrificans’ Metabolism
Source: PLoS One. 2014 Aug 29;9(8):e106218. doi: 10.1371/journal.pone.0106218 (PMC4149538; doi:10.1371/journal.pone.0106218)
Supplement: Table S1 — Growth properties and significant differences in distinct culture experiments. S. denitrificans were incubated under different conditions as listed in Figure 1. For selected time points p-values were calculated using the student’s t-test. (PDF) [file pone.0106218.s001.pdf]

Supplementary Information

Table S1: Growth properties and significant differences in distinct culture experiments.

S. denitrificans were incubated under different conditions as listed in Figure 1. For selected time points p-values were calculated using the student's t-test.

	+ H_2 + $S_2O_3^{2-}$ compared with + H_2 - $S_2O_3^{2-}$	+ H_2 - $S_2O_3^{2-}$ compared with + H_2 + $S_2O_3^{2-}$	+ H_2 + $S_2O_3^{2-}$ compared with - H_2 + $S_2O_3^{2-}$
growth description (p-value)	faster within first 3 days (0.003)	denser overall (0.002)	faster and denser (<0.001)
