# Supplementary material for: The Role of Hydrogen for Sulfurimonas denitrificans’ Metabolism
Source: PLoS One. 2014 Aug 29;9(8):e106218. doi: 10.1371/journal.pone.0106218 (PMC4149538; doi:10.1371/journal.pone.0106218)
Supplement: Table S2 — Significant differences of growth, hydrogen consumption and hydrogen uptake activity for cultures grown without thiosulfate (+H2−S2O3 2−) and cultures grown with thiosulfate (+H2+S2O3 2−). S. denitrificans were grown without thiosulfate (+H2−S2O3 2−) and with thiosulfate (+H2+S2O3 2−), the growth, H2 consumption and H2 uptake activity are shown in Figure 1, Figure 2 and Figure 5, respectively. Statistics were performed using the student’s t-test. (PDF) [file pone.0106218.s002.pdf]

Supplementary Information

Table S2: Significant differences of growth, hydrogen consumption and hydrogen uptake activity for cultures grown without thiosulfate (+ H_2 - $S_2O_3^{2-}$) and cultures grown with thiosulfate (+ H_2 + $S_2O_3^{2-}$).

S. denitrificans were grown without thiosulfate (+ H_2 - $S_2O_3^{2-}$) and with thiosulfate (+ H_2 + $S_2O_3^{2-}$), the growth, H_2 consumption and H_2 uptake activity are shown in Figure 1, Figure 2 and Figure 5, respectively. Statistics were performed using the student's t-test.

	denser growth overall	higher H_2 consumption	higher H_2 uptake activity
+ H_2 - $S_2O_3^{2-}$ compared with + H_2 + $S_2O_3^{2-}$	p-value 0.002	p-value 0.001	p-value <0.001
